# Antenatal corticosteroids and fetal lung immaturity in preterm birth

**DOI:** 10.1016/j.heliyon.2020.e04116

**Published:** 2020-06-18

**Authors:** Iyad Ali, Rita Imad Batta, Reem Mahmoud Yaseen, Jawad Hasson

**Affiliations:** a Faculty of Medicine and Health Science, An-Najah National University, Nablus, 707, Palestine; b Pediatrics Department, Rafidia Governmental Surgical Hospital, Nablus, Palestine

**Keywords:** Pathophysiology, Epidemiology, Public health, Respiratory system, Women's health, Pediatrics, Intensive care medicine, Laboratory medicine, Respiratory distress syndrome, Antenatal corticosteroids, Prematurity, Lung immaturity, Surfactant, Preterm birth

## Abstract

**Background:**

Respiratory distress syndrome (RDS), a consequence of lung immaturity, is a serious complication of preterm birth and the primary cause of early neonatal mortality. Administration of antenatal steroids is a standard care method for mothers with anticipated preterm labor. However, the gestational age range at which antenatal corticosteroids (ACS) provide benefit has been subjected to debate. This study aimed to find the prevalence of ACS use in patients that developed/did not develop RDS.

**Methods:**

This cross-sectional study was conducted at Rafidia governmental surgical hospital. It is based on the data obtained from the files of mothers who gave birth to premature babies and from a face-to-face interview. One hundred and twenty-eight data collection forms were completed over a period of seven months.

**Results:**

Approximately 64% of mothers, mothers who gave birth to premature babies, were given ACS, and about 33% of premature neonates developed RDS. Mothers who gave birth to newborns with RDS have lower odds of being administered ACS by 44% (OR = 0.44, CI = 0.202–0.94, p value = 0.034). However, the association became statistically not significant after adjusting gestational age, birth weight, gender, mother's age, intrauterine growth restriction (IUGR), mode of delivery and gestational hypertension (OR = 0.462, CI = 0.137–1.56, p value = 0.212). Higher risk of RDS was significantly associated with lower gestational age (p-value < 0.001) and IUGR after adjustment (p value = 0.035). However, no significant association could be found between RDS and mode of delivery (p value = 0.730), maternal age (p value = 0.63) and gender of the baby (p value = 0.22).

**Conclusions:**

the overall prevalence of RDS in preterm infants was 33.3% and the identified risk factors were lower gestational age and IUGR. We showed that the administration of ACS wasn't significantly associated with the development of RDS after adjusting gestational age, birth weight, gender, mother's age, IUGR, mode of delivery and gestational hypertension, as the administration of ACS didn't fully meet the international guidelines.

## 1 Introduction

Premature birth has a significant long-term health effect because of increased risk of death and the development of a wide range of chronic physical and neurological disabilities compared with long-term birth [1,2]. The frequency of preterm births is about 12–13% in the USA and 5–9% in many other developed countries; however, the rate of preterm birth has increased in many locations, predominantly because of increasing indicated preterm births and preterm delivery of artificially conceived multiple pregnancies [3]. Respiratory Distress Syndrome (RDS) is a serious complication of premature birth and the leading cause of early death and disability in newborns [4, 5, 6]. The main feature of RDS is the inadequate production of surfactant in the lungs of preterm infants due to the immature development of type II pneumocytes beginning with the production of surfactants after about 20 weeks of gestation [7]. Risk factors for this pathology include maternal age, birth weight, gestational age, elective care and emergency cesarean section (CS), gender, and prepartum asphyxia [8, 9, 10].

The administration of antenatal corticosteroids (ACS) to mothers at risk for premature birth is indicated to prevent RDS [11]. Limitations of available data are largely related to the low risk of severe airway morbidity after 34 weeks gestation and the lack of information on long-term outcome after corticosteroid exposure in late pregnancy [12, 13, 14, 15, 16, 17]. A single course of corticosteroids is recommended for pregnant women between 24 0/7 weeks and 33 6/7 weeks of gestation who are at risk of preterm delivery within 7 days, including for those with ruptured membranes and multiple gestations. It also may be considered for pregnant women starting at 23 0/7 weeks of gestation who are at risk of preterm delivery within 7 days, based on a family's decision regarding resuscitation, irrespective of membrane rupture status and regardless of fetal number [18]. The American College of Obstetricians and Gynecologists (ACOG) recommended giving a single course of corticosteroids to all pregnant women between 24 and 34 weeks of gestation who are at risk of preterm delivery within 7 days. Because of insufficient scientific evidence, the consensus panel also recommended that repeat corticosteroid courses, including so-called "rescue therapy," should not be routinely used but should be reserved for women enrolled in clinical trials. Betamethasone and dexamethasone have been most widely studied and have generally been the preferred corticosteroids for antenatal treatment to accelerate fetal organ maturation [19]. The non-compliance of the guidelines needs to be further investigated.

Prenatal corticosteroids in mothers with expected premature delivery improve survival, reduce the risk of RDS, necrotizing enterocolitis and intraventricular hemorrhage, and a single course does not appear to associate with significant adverse effects of the mother or child [20]. The optimal treatment interval is more than 24 h and less than 7 days after starting the steroid treatment, after 14 days, the benefit is lower [20]. The rates for prenatal corticosteroid use in low- and middle-income countries remain low [21], indicating that it is possible to improve the results for premature babies by administering ACS [22].

This study aimed to determine the prevalence of ACS use in patients that developed/did not develop RDS.

## 2 Methods

We performed this cross-sectional study for all newborns under the age of 37 weeks of gestational, born between August 1, 2016 and March 7, 2017, at the Rafidia Governmental Surgical Hospital (RGSH), the central governmental hospital in the north West Bank of Palestine. The ethical approval was obtained from the Institutional Review Board (IRB) at An-Najah National University. Informed consent was obtained from all participating mothers and they have been affirmed that their contribution is voluntary.

Participants in the study were the mothers who gave birth to premature babies. Mothers who received steroids for reasons other than pulmonary maturity of the fetus were excluded from the study. Dead newborns (stillbirths) have also been excluded, as death-finding investigations are not normally carried out in Palestine.

The demographic, antenatal, obstetric, medical and medical history and information on the administration of corticosteroids, route of administration, approval and evaluation in NICU, treatment and mortality were analyzed in clinical charts and analyzed face-to-face. The data collection form was prepared after a literature review [3,11,23,24]and taking into account the respondents and the type of analysis that should be used. The data collection form was reviewed by 10 medical specialties and revised accordingly. Subsequently, a pilot study was carried out with a sample of 15 participants and the data collection form reedited to its final form. The data collection form contained 23 questions, the first five questions related to age, education, residence, employment and consanguinity. The next section included 15 questions about prenatal period, and the last section consisted of 3 questions on previous pregnancies.

Gestational age is generally estimated as the difference between the first day of the last menstrual period and the date of delivery [25], and by ultrasound when a menstrual date is absent [26]. The diagnosis of RDS was based on a combination of clinical and radiographic features according to the criteria of Update on the European Consensus Guidelines on the Management of Neonatal Respiratory Distress Syndrome in Preterm Infants [20]. These criteria are: (1) PaO_2_ <50 mmHg or central cyanosis in the room air or additional oxygen demand to maintain PaO_2_> 50 mmHg or oxygen saturation> 85% within the first 24 h of life; and (2) chest radiography consistent with RDS (reticulogranular appearance for lung fields with or without low lung volume and air bronchograms) in the first 24 h of life [20].

The surfactant therapy Infasurf® (Kalfactant) was administered to newborns based on the NICU protocol for RGSH: an initial dose of 3 ml/kg and, if necessary (mean arterial pressure (MAP), remains >7 or FiO_2_ >35 %), a subsequent dose of 3 ml/kg is administered within 12 h.

Statistical analysis was performed with SPSS® for Windows® version 22. Continuous variables were characterized by mean (±standard deviation) or median (minimum-maximum) if they had symmetric or asymmetric distribution respectively, and categorical variables by absolute and relative frequencies. For comparison of continuous variables, parametric tests (independent t-test) or nonparametric tests (Mann-Whitney U-test) were used if they had two or more than two categories, and chi-square or Fisher's exact test for comparison categorical variables, the latter for expected values below 5. A p-value below 0.05 was considered statistically significant.

## 3 Results

One hundred and twenty eight mothers were included in our study. Mothers' characteristics are shown in Table 1. Eighty-two mothers (64.1%) were given ACS. Ten (11.7%) of mothers received ACS within less than 24 h of birth, another 55.9% had received ACS outside the 1-7-day period, while only 32.4% of them had received ACS within 24 h (Table 1).

**Table 1:** Mothers characteristic and their antenatal care.

Characteristics of mothers	Number (%)
**Education level**	
Elementary school	6 (4.7)
Middle school	29 (22.7)
High school	46 (35.9)
Diploma	15 (11.7)
Bachelor	32 (25.0)
**Employment**	
Employed	9 (7)
**Residency**	
City	33 (25.8)
Village	85 (66.4)
Camp	10 (7.8)
**Consanguinity**	
Consanguineous	28 (21.9)
**Antenatal corticosteroids**	
Given	82 (64.1)
≤34 weeks	45 (76.3)
>34 weeks	37 (53.6)
Not given	46 (35.9)
**Timing of ACS administration**	
Within <24 h of birth	10 (11.7)
Outside the 1-7-day period	46 (55.9)
According to the guidelines	32 (26)
**Place of follow up**	
Private sector	109 (85.2)
Governmental hospital	10 (7.8)
UNRWA∗	7 (5.5)
No follow up	2 (1.6)
**Detailed 20-week morphology scan**	
Done	84 (65.6)
Normal	73 (86.9)
Abnormal	11 (13.1)

The mean maternal age was 33.9 ± 2.5 weeks.

∗ United Nations Relief and Works Agency.

In our cross-sectional study, 45.3% of mothers had a normal BMI, only 9 mothers had gestational hypertension and 2 mothers had gestational diabetes. The premature rupture of the membrane was present in 22.7% of the mothers. Thirty-seven mothers (40.2%) had previous premature birth, 70% of women had previous pregnancy, 23.9% of mothers had an inter-pregnancy interval of less than one year, 29 mothers (22.7%) were multiparous, 15% of mothers had a history of babies with RDS and 99 mothers (77.3%) were pregnant with singleton (Table 2).

**Table 2:** Risk factors associated with the obstetric history of the mothers.

Risk factors	Number of mothers (%)
**Maternal pre-pregnancy BMI**	
Underweight <18.5	11 (8.6)
Healthy weight 18.5–24.9	58 (45.3)
Overweight 25–29.9	24 (18.8)
Obese ≥30	10 (7.8)
Missing data	25 (19.5)
**Gestational hypertension**	
Present	9 (7.0)
**Gestational diabetes**	
Present	2 (1.6)
**Premature rupture of membranes**	
Present	29 (22.7)
For more than 18 h	6 (20.7)
For less than 18 h	23 (79.3)
**Parity**	
Yes	114 (70.4)
**Previous preterm birth**	
Yes	37 (40.2)
**Inter-pregnancy interval**	
Less than 12 months	22 (23.9%)
More than 12 months	70 (76.1%)
**Multiple gestations**	
Yes	29 (22.7)
**Smoking**	
Smoker	19 (14.8)
**History of previous RDS in multiparous mothers**	17 (14.9)
**Gestation**	
Single	99 (77.3)
Twins	24 (18.8)
Triple	5 (3.9)

Newborn characteristics are summarized in (Table 3); about 54.3% of the newborns were males, the mean gestational age was 33.9 ± 2.5 weeks and mean birth weight was 2180.1 ± 656 g. Fifty-four (33.3%) newborns had RDS and most of newborns with RDS were aged ≤34 weeks of gestation. The mean age of the mothers was 27.2 ± 5.8 years.

**Table 3:** Characteristics of the newborns.

Characteristics of newborns	Number (%)
**Gestational age**	
Extremely preterm (<28 weeks' gestation)	2 (1.2)
Very preterm (<32 weeks' gestation)	29 (17.9)
Moderate preterm (32–34 weeks' gestation)	27 (16.7)
Late preterm (34–36 6/7 weeks' gestation)	104 (64.2)
**Mean= 33.9 ± 2.5 weeks**	
**Birth weight**	
Very low birth weight (<1500 g)	32 (19.8)
Low birth weight (1500–2500 g)	82 (50.6)
Normal birth weight (2500–4000 g)	46 (28.4)
Large for gestational age (>4000 g)	2 (1.2)
**Mean 2180.1 ± 656 gr**	
**RDS**	
Yes	54 (33.3)
≤34 weeks of gestation	48 (66.7)
>34 weeks of gestation	6 (6.7)
No	108 (66.7)
**Gender**	
Male	88 (54.3)
Female	74 (45.7)

Forty-one (75.9%) newborns with RDS required surfactant administration with a median of almost one dose (Table 4). Infants with RDS stayed significantly longer in neonatal intensive care units (NICU) (87% vs 41%, p < 0.0001) and had a significantly higher mortality rate (25.9% vs 2.1%, p = 0.001) compared to those who did not have RDS. The majority of newborns with RDS had no other complications.

**Table 4:** Evaluation of newborns with RDS at the NICU.

Newborn babies with RDS (n-54)	Number (%)
**Given Surfactant**	
Yes	41 (75.9)
Stay in NICU (days)	47 (87)
**Outcome**	
Died	14 (25.9)
Transferred	1 (1.9)
Discharged home (treated)	39 (72.2)

**Complications**	
No complications	43 (79.6)
Complications	11 (20.4)
Pulmonary hemorrhage	4 (7.4)
Necrotizing entero-colitis	1 (1.9)
Bronchopulmonary dysplasia	1 (1.9)
Pneumothorax	1 (1.9)
Disseminated intravascular coagulation	2 (3.7)
Meningitis	2 (3.7)

Female newborns developed RDS less frequently than males (Table 5). However, the association was not significant even after the adjustment of gestational age, birth weight, ACS, maternal age, intrauterine growth restriction (IUGR), delivery method and pregnancy hypertension using the logistic regression test. The neonates who developed RDS had a significant lower gestational age and birth weight compared to gestational age (p value < 0.0001) and birth weight (p value < 0.0001) of those who did not develop RDS (Table 5).

**Table 5:** The effect of risk factors in the presence of RDS among newborns.

Risk factors for RDS	Total	RDS	Without RDS	P value
**Gender, n (%)**				
Male	88 (100)	33 (37.5)	55 (62.5)	0.212^1^
Female	74 (100)	21 (28.4)	53 (71.6)	
Gestational age (weeks), median (min-max)	33.9 (25.1–36.9)	31.6 (25.1–35.7)	35 (28–36.9)	<0.001³
Birth weight (grams), mean (±SD)	2180.1 (656.7)	1769.3 (673.4)	2385.5 (544.5)	<0.001^2^
Antenatal corticosteroids, n (%)	111 (68.5)	43 (38.7)	68 (61.3)	0.034^1^
Antenatal corticosteroid administration timing by gestational age, mean (±SD)	30.8 (3.1) week	29.7 (2.7) week	31.5 (3.2) week	0.002^2^
Time between the last dose of a full cycle of steroids and delivery (days), mean	16.5	13.9	18.2	0.499³
Intrauterine growth restriction, n (%)	8 (4.9)	1 (1.9)	7 (6.5)	0.200^4^
Pregnancy complications, n (%)				
Gestational diabetes	2 (1.2)	1 (1.9)	1 (0.9)	1.00⁴
Gestational hypertension	9 (5.6)	2 (3.7)	7 (6.5)	0.719⁴
Premature rupture of membranes	38 (23.5)	5 (9.3)	33 (30.6)	0.003^1^
**Mode of delivery, n (%)**				
Vaginal	60 (37)	19 (35.2)	41 (38)	0.730^1^
C-section	102 (63)	35 (64.8)	67 (62)	

^1^Chi-square test, ^2^Independent T-test, ³Mann-Whitney U-test, ⁴Fisher-exact test.

The gestational age at which prenatal corticosteroid was administered was lower in mothers whom newborns developed RDS than mothers whom newborns didn't develop RDS (t = 3.144, p-value = 0.002). However, there was no statistically significant time-difference between the last dose of a full steroid cycle and the delivery (Table 5).

Univariate analysis confirmed that mothers giving birth to newborns with RDS are less likely to get ACS (OR = 0.44, CI = 0.202–0.94, p-value = 0.034). However, the association did not become statistically significant after adjusting gestational age, birth weight, gender, maternal age, IUGR, type of birth, and gestational hypertension (p-value = 0.212) (Table 6). Although the association between RDS and gestational age remained significant (p-value <0.0001), after adjustment of birth weight, gender, maternal age, IUGR, delivery mode, and gestational hypertension (Table 6), however, the association between RDS and birth weight (p-value = 0.0001) disappears after adjustment of gestational age, gender, maternal age, IUGR, delivery mode, and gestational hypertension (p-value = 0.43).

**Table 6:** Unadjusted and adjusted values using logistic regression.

Variables	OR (CI)	P- value	Adjusted∗ OR (CI)	P-value
ACS	0.44 (0.202–0.94)	0.034	0.462 (0.137–1.56)	0.212
GA	0.42 (0.33–0.55)	<0.01	0.338 (0.225–0.508)	<0.0001
IUGR	0.673 (0.44–30.65)	0.229	23.499 (1.256–439.79)	0.035
Mother age	1.014 (0.957–1.075)	0.63	0.958 (0.869–1.056)	0.389
Mode of delivery	1.127 (0.571–2.226)	0.73	2.117 (0.672–6.674)	0.2

∗ adjusted for maternal age, gestational hypertension, gender, mode of delivery, birth weight, gestational age (GA), intrauterine growth restriction (IUGR) and antenatal corticosteroids (ACS). OR: odds ratio, IC: confidence interval.

There was no statistically significant association between RDS and IUGR, maternal age or mode of delivery (Table 6). The association between RDS and IUGR became significant after adjustment (p-value = 0.035).

## 4 Discussion

The overall prevalence of RDS in preterm infants in our study was about 33.3%. The prevalence of RDS in preterm babies born at or before 34 weeks of gestation was higher than other studies [27]. Also, the prevalence of RDS in those born after 34 weeks of gestation is slightly higher than reported in other studies as well [23]. Prenatal corticosteroid therapy to improve fetal lung maturity has become an established treatment modality [28]. Approximately 76.3% of mothers who had given birth before or at 34 weeks of gestation had received prenatal corticosteroids, which is slightly lower than the average use of prenatal corticosteroids in the US and many other high-income countries [29]. Based on high-grade evidence, antenatal steroid therapy is very effective in preventing neonatal mortality and morbidity, yet remains at low coverage in low/middle-income countries [30]. An interesting finding was that the administration of prenatal corticosteroids after adjustment of gestational age and birth weight was not significantly associated with the prevention of RDS, although this was significantly associated with a decreased risk of RDS prior to adjustment. This may be due to improper compliance with ACS administration guidelines of the ACOG [19].

The time between the last dose of a full cycle of prenatal corticosteroids and childbirth was not significantly associated with the development of RDS in preterm infants, which is incompatible with many studies suggesting that the optimal treatment interval is more than 24 h and less than 7 days after the start of steroid treatment, and the benefit is reduced beyond 14 days [20]. This can be explained by the fact that the deviations of the administration time in our study occurred outside the period of 1–7 days stated in the guidelines. In our study, a lower gestational age was associated with an increased risk of RDS, in consistent with existing literature [8,10]. Neither male gender nor lower birth weight was significantly associated with an increased risk of RDS in contrast to previous studies [8,10]. Also, the age of the mother was not significantly associated with RDS in consistent with one study [10] but contradicts with another [8]. In contrast to other studies that indicated an increased risk of RDS in cesarean delivery compared to normal vaginal delivery, the mode of delivery was not significantly associated with RDS in our study [8,10]. Surfactant administration was needed in 41 (75.9%) newborns with RDS, with a median of nearly one dose (1.39), in agreement with previous study [27]. As expected, the mortality rate was significantly higher in newborns with RDS, and out of 15 deceased newborns, 14 (25.9%) had RDS.

This is the first study to investigate the prevalence of RDS in Palestine and the rate of prenatal corticosteroids administration. In addition, this study focused on neonates who were delivered through all preterm gestational ages. The weakness of this study was its observational nature, the small sample size and a single hospital-based data collection. In addition, the information documented in the patient medical files was not properly completed and the coincidence that most cases were of late prematurity.

## 5 Conclusions

The overall prevalence of RDS in preterm infants was 33.3% and the identified risk factors were lower gestational age and IUGR. We have shown that the administration of a complete cycle of prenatal corticosteroids after adjustment is not significantly related to the development of RDS as the administration were not fully complied with ACOG guidelines. Further studies may be helpful to evaluate the factors hindering the implementation of ACS administrative guidelines.

## Declarations

### Author contribution statement

I. Ali, R. Batta and R. Yaseen: Conceived and designed the experiments; Performed the experiments; Analyzed and interpreted the data; Wrote the paper.

J. Hasson: Conceived and designed the experiments; Performed the experiments.

### Funding statement

This work was supported by An-Najah National University, Nablus, Palestine.

### Competing interest statement

The authors declare no conflict of interest.

### Additional information

No additional information is available for this paper.
